# In search of novel PD1 inhibitor from natural products by high-throughput virtual screening and molecular dynamics simulation

**DOI:** 10.1371/journal.pone.0339355

**Published:** 2026-04-22

**Authors:** Neha Sharma, Abhijit Debnath, Rupa Mazumder, Pallavi Rai, Rajesh Kumar Singh

**Affiliations:** 1 Noida Institute of Engineering and Technology (Pharmacy Institute), Uttar Pradesh, India; 2 Ram-Eesh Institute of Vocational and Technical Education, Uttar Pradesh, India; 3 Department of Dravyaguna, Institute of Medical Sciences, Banaras Hindu University, Varanasi-, India; Chung-Ang University, KOREA, REPUBLIC OF

## Abstract

Squamous cell carcinoma (SCC) represents a significant oncological challenge. While immune checkpoint inhibitors targeting PD-1/PD-L1 have revolutionized the treatment of SCC, current monoclonal antibody approaches face limitations, including poor tissue penetration, high costs, and immune-related adverse events in patients. Most existing small-molecule efforts target PD-L1, leaving PD-1/PD-L2 interactions intact and enabling immune escape. This study represents the first systematic identification of natural product-derived direct PD-1 inhibitors, offering broader pathway blockade compared to PD-L1-selective approaches. While current therapeutic limitations highlight the need for alternative methods, this computational study lays a foundation for experimental validation and potential advancement of a drug development pipeline. Through integrated computational screening of 17,967 phytochemicals from the IMPPAT database, we employed consensus molecular docking across seven algorithms, 300-ns molecular dynamics simulations, density functional theory calculations, and comprehensive ADME profiling. IMPHY004834 (Mahuannin D) from *Ephedra sinica* emerged as a lead compound with exceptional free binding energy, forming stable interactions with key PD-1. Molecular dynamics analysis revealed remarkable stability with consistent RMSD, lowest RMSF, and sustained hydrogen bonding throughout the simulation. The biflavonoid structure exhibits a favorable HOMO-LUMO gap, indicating chemical stability, while ADME profiling confirms drug-like properties, albeit requiring parenteral administration due to low GI absorption. This work establishes the first evidence for Mahuannin D’s PD-1 inhibitory mechanism, which was previously known only for its cytotoxic effects. It provides a validated computational framework for discovering natural product-based immune checkpoint inhibitors with superior pathway coverage compared to existing PD-L1-selective therapeutics.

## 1. Introduction

Squamous cell carcinoma (SCC) represents a formidable clinical challenge in oncology, constituting the second most prevalent form of non-melanoma skin cancer with an estimated annual incidence exceeding 1 million cases globally [1,2]. This malignancy arises from the aberrant proliferation of keratinocytes within the stratified squamous epithelium, demonstrating considerable heterogeneity in its clinical presentation, biological behavior, and therapeutic response [3,4]. While the majority of cutaneous squamous cell carcinomas (cSCC) present as localized lesions amenable to surgical intervention, a clinically significant subset exhibits aggressive characteristics, including rapid growth, deep tissue invasion, perineural involvement, and propensity for regional and distant metastasis [5]. The established risk factors encompass chronic ultraviolet radiation exposure, immunosuppressive states, human papillomavirus infection, chronic inflammatory conditions, and specific genetic predispositions, collectively contributing to the multifactorial pathogenesis of this neoplasm [6]. The contemporary therapeutic armamentarium for SCC encompasses a multimodal approach tailored to disease stage, anatomical location, and patient-specific factors. Surgical excision with histologically confirmed negative margins remains the cornerstone of treatment for localized disease, achieving cure rates exceeding 95% in appropriately selected cases [4]. However, advanced, recurrent, or metastatic SCC presents substantial therapeutic challenges, necessitating aggressive interventions including wide local excision, lymph node dissection, radiation therapy, and systemic chemotherapy [7]. These conventional therapeutic modalities are frequently associated with significant morbidity, including disfigurement, functional impairment, and dose-limiting toxicities that compromise quality of life and therapeutic efficacy [8]. Furthermore, the emergence of drug resistance, particularly in advanced disease, underscores the urgent need for novel therapeutic strategies that can overcome the limitations of existing treatments.

The tumor microenvironment in SCC is characterized by complex interactions between malignant cells, stromal components, and immune effectors, with mounting evidence demonstrating the critical role of immune surveillance in disease progression and therapeutic response [9,10]. Cancer cells have evolved sophisticated mechanisms to evade immune recognition and destruction, prominently featuring the exploitation of immune checkpoint pathways that generally serve to maintain immune homeostasis and prevent autoimmunity [11]. Among these regulatory mechanisms, the Programmed Death-1 (PD-1)/Programmed Death-Ligand 1 (PD-L1) axis has emerged as a pivotal immune checkpoint pathway that significantly influences anti-tumor immunity [12]. PD-1, a 55-kDa type I transmembrane glycoprotein belonging to the immunoglobulin superfamily, is predominantly expressed on activated T cells, B cells, natural killer cells, and myeloid cells [13]. Upon engagement with its cognate ligands PD-L1 (B7-H1/CD274) and PD-L2 (B7-DC/CD273), PD-1 initiates downstream signaling cascades that involve the recruitment of phosphatases SHP-1 and SHP-2, subsequently leading to the dephosphorylation of key signaling molecules in the T cell receptor pathway [14]. This molecular interaction culminates in T cell anergy, exhaustion, and apoptosis, effectively dampening the immune response [15,16]. In the context of cancer, tumor cells and tumor-associated stromal cells frequently overexpress PD-L1 as an adaptive immune resistance mechanism, creating an immunosuppressive microenvironment that facilitates tumor progression and metastasis [15,16].

**Fig 1:**
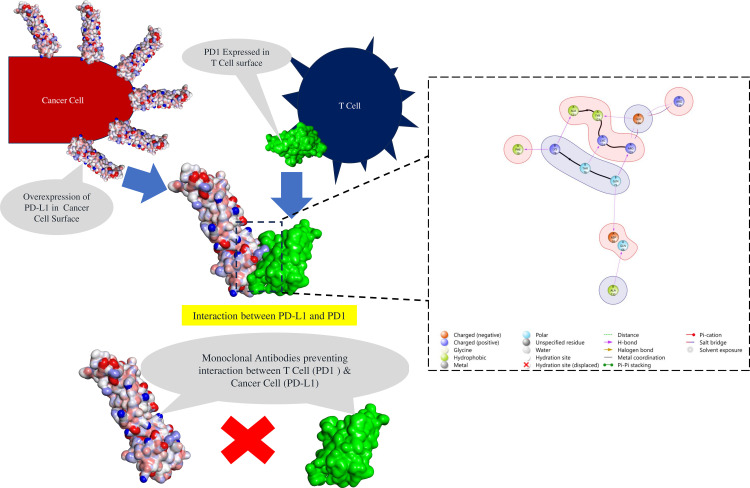
Illustration of PD1-PDL1 interaction (PDB id: 4ZQK) and mechanism of Action of PD-1 Inhibitors.

At the molecular level, the PD-1/PD-L1 interaction represents a well-characterized protein-protein interface critical for immune checkpoint regulation (Fig 1). Structural analysis of the PD-1/PD-L1 complex reveals a binding interface spanning approximately 1,970 Å², characterized by a network of strategic interactions involving both hydrophobic and polar contacts. The primary binding interface on PD-1 involves several critical residues that form the foundation of PD-L1 recognition. Key PD-1 residues include Asn66, Tyr68, Thr76, Asp77, Lys78, Gly124, Ile126, Ser127, Lys131, Ala132, Gln133, Ile134, and Glu136, which collectively create a binding surface that accommodates PD-L1 through multiple interaction modes. Specifically, Asn66 forms critical contacts with PD-L1’s Gln66, Tyr123, Ala121, and Asp122, while Tyr68 engages in essential interactions with PD-L1’s Tyr123. The Thr76 residue contributes significantly through hydrogen bonding with PD-L1’s Lys124, and Lys78 establishes multiple contacts, including hydrogen bonds with PD-L1’s Phe19 and Ala121. Hydrophobic interactions play a crucial stabilizing role, with Ile126 and Ile134 forming extensive van der Waals contacts with PD-L1’s Met115, Tyr123, and Ala121. The Glu136 residue contributes through dual functionality, forming both hydrogen bonds and salt bridges with PD-L1’s Arg113 and Arg125, demonstrating the electrostatic complementarity essential for stable complex formation [17–21].

The clinical validation of immune checkpoint inhibition has revolutionized cancer therapeutics, with monoclonal antibodies targeting the PD-1/PD-L1 axis demonstrating remarkable efficacy across diverse malignancies [22]. In SCC, pembrolizumab and cemiplimab, both anti-PD-1 monoclonal antibodies, have shown significant clinical activity in advanced and metastatic disease, leading to their regulatory approval for these indications [23,24]. However, despite these therapeutic advances, monoclonal antibody-based immunotherapies are constrained by several inherent limitations that potentially compromise their clinical utility and accessibility. These include substantial manufacturing costs leading to prohibitive pricing, requirement for intravenous administration necessitating specialized healthcare infrastructure, limited tissue penetration due to large molecular size, potential immunogenicity resulting in neutralizing antibody formation, and complex cold-chain storage requirements [25,26]. Moreover, immune-related adverse events (irAEs) associated with checkpoint inhibitor therapy can affect virtually any organ system, manifesting as pneumonitis, colitis, hepatitis, endocrinopathies, dermatologic toxicities, and rare but potentially fatal neurologic complications [27,28]. These toxicities, while generally manageable with appropriate monitoring and immunosuppressive interventions, can result in treatment discontinuation and long-term sequelae that significantly impact patient outcomes and quality of life [29]. The limitations of monoclonal antibody-based therapeutics have catalyzed intensive research efforts toward the development of small-molecule inhibitors targeting the PD-1/PD-L1 interaction [28]. Small-molecule checkpoint inhibitors offer several theoretical advantages over their antibody counterparts, including oral bioavailability enabling convenient outpatient administration, superior tissue penetration facilitating enhanced tumor targeting, reduced immunogenicity minimizing the risk of neutralizing antibody formation, cost-effective synthesis and manufacturing, simplified storage and distribution logistics, and potential for combination with other therapeutic modalities [30,31].

Beyond monoclonal antibodies and small-molecule approaches, peptide-based vaccines targeting immune checkpoints represent an emerging and promising strategy for cancer immunotherapy. These vaccines aim to stimulate the host’s immune system to produce endogenous antibodies against PD-1, thereby blocking its interaction with PD-L1 and enhancing T-cell activation through active immunization rather than passive antibody [32]. Several innovative vaccine platforms have been developed, including PD1-Vaxx, which consists of a PD-1 B cell peptide (mimotope) linked to a measles virus fusion peptide, designed to induce antibodies that functionally mimic therapeutic monoclonal antibodies while providing immunological memory [33,34]. Combined vaccination strategies targeting multiple immune checkpoints simultaneously, such as dual vaccination approaches against both PD-1 and tumor-associated antigens like Her-2/neu, have shown synergistic effects in preclinical models [35]. Computational biology approaches are also advancing vaccine development through in silico design and optimization of peptide-based immunogens, enabling rational vaccine design and epitope prediction [36]. These peptide-based vaccines offer several theoretical advantages over monoclonal antibodies, including the potential for long-lasting immunological memory, reduced manufacturing costs, potential for oral delivery, and the ability to overcome tumor escape mechanisms through polyclonal antibody responses. However, safety considerations regarding sustained immune activation against self-antigens and the potential for autoimmune reactions remain important factors requiring careful evaluation in clinical development [32,36].

Natural products have historically served as an invaluable source of pharmacologically active compounds, contributing to approximately 60% of currently approved anticancer therapeutics either directly or as synthetic derivatives [33–35]. The structural diversity inherent in natural product libraries, shaped by millions of years of evolutionary pressure, provides access to unique chemical scaffolds that may not be readily accessible through conventional synthetic chemistry approaches [33,37]. These compounds often exhibit favorable drug-like properties, including appropriate molecular weight, lipophilicity, and hydrogen bonding capacity, while demonstrating specific biological activities that can be optimized through medicinal chemistry efforts. Recent advances in computational drug discovery methodologies, including structure-based virtual screening, molecular dynamics simulations, and artificial intelligence-driven approaches, have significantly accelerated the identification of bioactive natural products with therapeutic potential [38]. High-throughput virtual screening platforms enable the rapid evaluation of extensive chemical libraries against specific molecular targets. In contrast, sophisticated molecular modeling techniques provide detailed insights into protein-ligand interactions, binding kinetics, and structure-activity relationships [39]. The IMPPAT (Indian Medicinal Plants, Phytochemistry, and Therapeutics) database represents a comprehensive repository of phytochemical information, containing detailed structural and pharmacological data for thousands of natural compounds derived from traditional medicinal plants [40,41]. This curated database offers an excellent starting point for discovering novel bioactive molecules with potential therapeutic applications in various disease contexts. Given the urgent clinical need for improved therapeutic options in SCC management and the inherent limitations of current immunotherapeutic approaches, the present study was designed to systematically identify and characterize novel small-molecule PD-1 inhibitors from natural product sources. Through an integrated computational pipeline encompassing structure-based virtual screening of the IMPPAT database, consensus molecular docking using multiple algorithmic approaches, density functional theory calculations for electronic property analysis, extensive molecular dynamics simulations, and comprehensive ADME profiling, we aimed to discover natural product-derived compounds with potent PD-1 inhibitory activity and favorable pharmacological properties.

This multidisciplinary approach leverages cutting-edge computational methodologies to bridge the gap between traditional natural product chemistry and modern drug discovery, potentially yielding novel therapeutic candidates that combine the structural diversity of natural products with the mechanistic precision of targeted immunotherapy. The identification of such compounds could provide the foundation for the development of next-generation immune checkpoint inhibitors that overcome the current limitations of antibody-based therapeutics while maintaining or enhancing their clinical efficacy in SCC and other malignancies.

## 2. Experimental

### 2.1. Identification of hits by structure-based virtual screening

#### 2.1.1. Receptor preparation and active site.

The crystal structures of PD-1 previously obtained from RCSB-PDB have been obtained and subjected to analysis. Among the 24 structures examined, a single structure (PDB id: 6j14, chain C) was chosen due to its superior resolution and comprehensive coverage of the entire sequence, encompassing the RBD area. The UCSF Chimera program’s Dockprep module [42] has been used to remove the antibody attached to it, compute Kohlmann charges, and subsequently introduce polar hydrogen atoms. The active site and roster of pivotal amino acid residues (Asn66, Tyr68, Thr76, Asp77, Lys78, Gly124, Ile126, Ser127, Lys131, Ala132, Gln133, Ile134, Glu136) that engage with ligands of these macromolecules have been derived from the literature [17–20,33–35,37–40] and subsequently verified using CASTp [43] and AADS [44]. The central coordinates of the grid box are [X = −17.503304, Y = −0.356822, Z = 5.444953], and the grid dimensions are 17 x 17 x 17.

#### 2.1.2. Ligand library preparation.

IMPPAT [41] is a manually curated database that catalogs 4010 Indian medicinal plants, their 17967 phytochemicals, to support cheminformatics-driven drug. All 17967 phytochemicals available in the IMPAAT v2.0 database have been downloaded individually in 3D-pdbqt format. The structures have been thoroughly examined for any potential errors. Subsequently, hydrogen atoms are introduced into the structures using the RDkit [45], and the resulting structures are converted into PDBQT format using Open Babel [46] followed by the preparation of ligands by using prepare_ligand4.py of AutoDockTools. A thorough search has been carried out with QuickVina-W [47]. A threshold of −6.0 kcal/mol of free binding energy has been set as a cut-off. Phytochemicals that meet the criteria have been selected for Consensus Molecular Docking. The threshold was set as per a reported similar study by Ting-ting Li et. al [48].

### 2.2. Consensus molecular docking

Consensus molecular docking is preferred over single-software docking because it combines results from multiple docking programs, thereby reducing software-specific biases and providing a more reliable prediction of binding interactions by leveraging the strengths of different algorithms and scoring functions [49,50]. When multiple docking programs consistently identify strong binding interactions for a particular compound, it increases confidence in the results. It helps filter out false positives that might score well in one program but poorly in others, ultimately leading to more robust and trustworthy predictions [51]. The consensus molecular docking has been done by following the same protocol we have followed in our previous works [52,53] where 7 different molecular docking programs have been used AutoDock Tools 4.2 [54], idock [55], Ledock [56], Qvina 2 [57], Smina [58], Vina [59,60], and PLANTS [61]. To obtain a balanced assessment of a compound’s performance across multiple docking algorithms, the Rank-by-Rank (RbR) method was used to calculate the final rank of each molecule. The ranking is based on the area of each molecule’s radar plot, where each axis represents a different docking method. A smaller radar plot area indicates better overall performance across all docking methods, as it means the molecule consistently ranked well (closer to the center) in multiple methods. Molecules are then ranked from best to worst based on their radar plot areas, with the smallest area receiving the top rank. The goal was to obtain a more robust ranking of potential PD-1 inhibitors derived from natural products. This consensus approach helps account for the inherent limitations and biases of individual docking algorithms, potentially leading to more reliable predictions of binding modes.

### 2.3. DFT calculation

Molecular structures have been evaluated using DFT calculations to assess their reactivity and stability. The workflow encompassed the conversion of molecular structures into XYZ format files with Open Babel [46], while also detailing the computational methodologies and basis sets employed. The ORCA software [62] DFT calculations have been performed using this method, and the resulting outputs have been saved for analysis. To facilitate the viewing and investigation of molecular orbitals and electronic structure, the outputs have subsequently been transformed into Molden format. IboView [63,64] has been used to exhibit electrical characteristics from the Molden input format.

### 2.4. Molecular dynamics simulation

Conformational dynamics and stability assessment of protein-ligand complexes were conducted through all-atom molecular dynamics simulations utilizing GROMACS 2022.4 over a 300-nanosecond trajectory [65], following previously established computational protocols [53,66]. The simulation framework incorporated the CHARMM36 all-atom force field (July 2022 version) [67,68] for protein parameterization and CGenFF-derived parameters [69,70] for ligand molecules, with the complete system solvated using the TIP3P water model under periodic boundary conditions [71–73]. The computational workflow encompassed initial energy minimization via steepest descent algorithm (maximum force criterion: 1000 kJ/mol/nm), followed by sequential equilibration phases including NVT ensemble (100 ps duration at 300 K employing V-rescale thermostat with τ = 0.1 ps) and NPT ensemble (100 ps at 300 K and 1 bar using Parrinello-Rahman barostat with τ = 2.0 ps), culminating in production runs of 500 ns with two fs integration time steps. Technical specifications included the LINCS algorithm implementation for hydrogen bond constraints, the Particle Mesh Ewald methodology for long-range electrostatic calculations (with a 1.2 nm cutoff), van der Waals interaction truncation at 1.2 nm with smooth switching initiated at 1.0 nm, and coordinate sampling intervals of 10 ps. Computational execution was performed on the PARAM Shivay high-performance computing infrastructure, with subsequent trajectory analysis encompassing RMSD, RMSF, Rg, SASA, PCA, FEL, and hydrogen bonding dynamics using integrated GROMACS analytical tools and MDAnalysis, NGLView, Pandas, and Matplotlib [74–77], thereby providing comprehensive characterization of structural stability, conformational flexibility, and intermolecular interaction patterns under physiologically relevant simulation conditions. The g_mmpbsa [78] was used to calculate Free binding energy by employing the last five ns of trajectory data for statistical analysis.

### 2.5. ADME

Drug development faces significant attrition due to suboptimal ADMET properties, with approximately 40% of candidate compounds failing in the preclinical stages primarily due to inadequate pharmacokinetics [79]. SwissADME is utilized to predict and optimize ADMET properties [80].

## 3. Results

### 3.1. Identification of hits by structure-based virtual screening

After completion of Structure-based virtual screening (SBVS) of the IMPAAT database, 31 hit molecules have been found with binding energy less than −7.0 kcal/mol, shown in S1 Material. All 31 molecules have been taken for Consensus Molecular Docking for further studies.

### 3.2. Consensus molecular docking

Compared to single docking methods, consensus docking by employing multiple docking programs has enhanced the quality of docking and virtual screening results with high accuracy [81–83]. The results of molecular docking, as determined by multiple docking programs, are presented in Table 1.

**Table 1:** Consensus molecular docking results for 31 phytochemicals against PD-1 (PDB ID: 6J14). Binding energies (kcal/mol) and PLANTS SCORE obtained from seven different docking algorithms: AutoDock Tools (ADT), iDock, LeDock, Qvina2, Smina, Vina, and PLANTS.

Molecule ID	ADT	iDock	LeDock	Qvina	Smina	Vina	PLANTS SCORE
	binding energy in Kcal/mol						
IMPHY000749	−5.8	−5.63	−4.29	−5.5	−6.36168718	−5.516	−23.1232
IMPHY001112	−9.01	−6.53	−4.24	−6.4	−6.56255293	−6.485	−23.3898
IMPHY001137	−7.27	−5.7	−3.12	−5.5	−5.51428986	−5.576	−19.641
IMPHY002235	−8.18	−5.41	−3.62	−5.3	−5.54429579	−5.353	−19.9147
IMPHY002354	−7.76	−6.43	−3.84	−6.3	−6.31967449	−6.354	−23.4891
IMPHY003213	−9.36	−7.15	−4.72	−7	−7.80591774	−7.029	−25.2588
IMPHY003388	−10.78	−7.75	−5.99	−7.6	−8.89089966	−7.639	−30.5046
IMPHY003529	−9.77	−6.62	−4.59	−6.5	−6.87833834	−6.423	−25.5034
IMPHY004244	−9.24	−6.16	−4.1	−6.1	−6.33682346	−6.143	−22.2548
IMPHY004515	−8.79	−7.1	−4.54	−6.8	−7.44745255	−6.917	−26.2779
IMPHY004657	−8.23	−6.73	−4.35	−6.6	−6.57699776	−6.539	−23.3057
IMPHY004740	−10.8	−5.86	−5.48	−5.6	−6.49073839	−5.455	−30.8556
IMPHY004834	−10.26	−6.89	−5.5	−6.7	−7.69255066	−6.724	−32.6407
IMPHY004879	−8.12	−5.52	−5.04	−5.4	−6.0210104	−5.457	−24.3221
IMPHY005033	−8.41	−6.9	−4.91	−6.7	−7.57105207	−6.753	−26.9581
IMPHY006072	−9.32	−6.03	−4.37	−5.9	−6.21307039	−5.822	−24.5431
IMPHY006688	−8.38	−6.44	−3.84	−6.4	−6.4461484	−6.464	−22.3314
IMPHY006882	−7.51	−7.48	−4.09	−7.3	−7.28467226	−7.299	−22.5238
IMPHY006927	−7.33	−6.86	−4.08	−6.7	−6.67527962	−6.648	−26.4132
IMPHY007665	−8.22	−6.89	−4.64	−6.7	−7.18536901	−6.641	−25.8599
IMPHY010091	−7.14	−7.04	−3.64	−6.9	−6.87120056	−6.905	−21.443
IMPHY011624	−8.89	−5.72	−3.66	−5.6	−5.78597546	−5.649	−23.5078
IMPHY012536	−11.55	−7.14	−5.42	−7.2	−7.66504097	−7.226	−28.6494
IMPHY013006	−7.41	−6.4	−3.48	−6.2	−6.41249418	−6.23	−24.9737
IMPHY013015	−7.72	−6.84	−4.18	−6.8	−6.9606986	−6.609	−26.4065
IMPHY013285	−9.54	−6.49	−4.84	−6.3	−7.00919008	−6.363	−28.2672
IMPHY013601	−8.98	−6.81	−3.92	−6.6	−6.80245543	−6.657	−24.6636
IMPHY014026	−3.5	−3.66	−5.35	−3.5	−2.1	−3.4	−20.11
IMPHY014076	−12.36	−6.84	−5.35	−6.8	−8.01279831	−6.555	−28.1998
IMPHY014146	−7.91	−7.24	−4.27	−7.1	−7.05308867	−7.168	−26.5452
IMPHY014498	−12.23	−8.25	−5.54	−8	−8.70506573	−8.038	−31.58
IMPHY014981	−7.76	−6.37	−3.77	−6.2	−6.22190332	−6.254	−21.5916

Numerous studies have demonstrated the efficacy of the Rank-by-rank (RbR) technique in optimizing outcomes through consensus molecular docking [49,84]. The scores assigned to the collected values are assigned ranks individually, with a rank of 1 indicating the best beneficial outcome. To facilitate the comparison of the placements of molecules across all four functions, the ranks of each scoring function are aggregated. Table 2 displays the final unified rating for each molecule, calculated by averaging its rankings across the scoring functions.

**Table 2:** Rank-by-rank consensus scoring results for molecular docking analysis. Individual rankings from seven docking algorithms were combined using radar plot area calculations, where smaller areas indicate better overall performance. Final ranking represents consensus performance across all methods, with rank 1 being the best. Top 6 compounds (highlighted) were selected for further analysis.

Molecule ID	ADT Ranking	iDock Ranking	LeDock Ranking	Qvina Ranking	Smina Ranking	Vina Ranking	PLANTS Ranking
IMPHY000749	31	29	17	28	23	28	24
IMPHY001112	12	18	19	18	19	17	22
IMPHY001137	29	28	32	28	31	27	32
IMPHY002235	20	31	30	31	30	31	31
IMPHY002354	23	21	25	20	25	21	21
IMPHY003213	9	5	11	6	4	6	15
IMPHY003388	5	2	1	2	1	2	4
IMPHY003529	7	17	13	17	14	19	14
IMPHY004244	11	24	21	24	24	24	27
IMPHY004515	15	7	14	8	8	7	12
IMPHY004657	18	16	16	15	18	16	23
IMPHY004740	4	26	4	26	20	30	3
IMPHY004834	6	10	3	11	5	10	1
IMPHY004879	21	30	8	30	28	29	19
IMPHY005033	16	9	9	11	7	9	8
IMPHY006072	10	25	15	25	27	25	18
IMPHY006688	17	20	25	18	21	18	26
IMPHY006882	26	3	22	3	9	3	25
IMPHY006927	28	12	23	11	17	12	10
IMPHY007665	19	10	12	11	10	13	13
IMPHY010091	30	8	29	7	15	8	29
IMPHY011624	14	27	28	26	29	26	20
IMPHY012536	3	6	5	4	6	4	5
IMPHY013006	27	22	31	22	22	23	16
IMPHY013015	25	13	20	8	13	14	11
IMPHY013285	8	19	10	20	12	20	6
IMPHY013601	13	15	24	15	16	11	17
IMPHY014026	32	32	6	32	32	32	30
IMPHY014076	1	13	6	8	3	15	7
IMPHY014146	22	4	18	5	11	5	9
IMPHY014498	2	1	2	1	2	1	2
IMPHY014981	23	23	27	22	26	22	28

**Fig 2:**
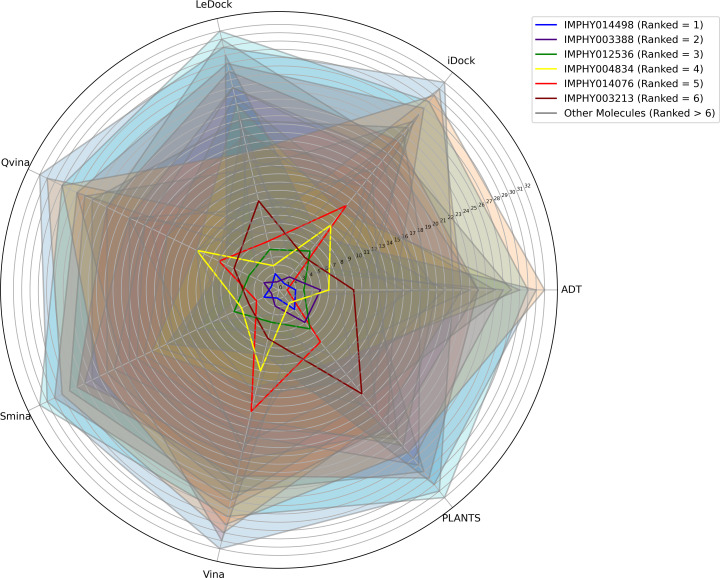
Radar plot visualization of consensus molecular docking results using Rank-by-Rank (RbR) methodology. Each axis represents a ranking from different docking algorithms. Smaller radar plot areas indicate better consensus performance across multiple algorithms. The top six compounds (IMPHY014498, IMPHY003388, IMPHY012536, IMPHY004834, IMPHY014076, IMPHY003213) showing the smallest areas were selected for subsequent DFT and MD analysis.

The final ranking provides a comprehensive assessment of the compounds’ anticipated effectiveness in binding, incorporating their ranks across various scoring techniques. The six most prominent compounds (IMPHY014498, IMPHY003388, IMPHY012536, IMPHY004834, IMPHY014076, IMPHY003213) that constitute the collective results of RbR have been chosen for subsequent investigations. These molecules have been graphically represented in the radar plot and are depicted in Fig 2.

### 3.3. DFT calculation

**Fig 3:**
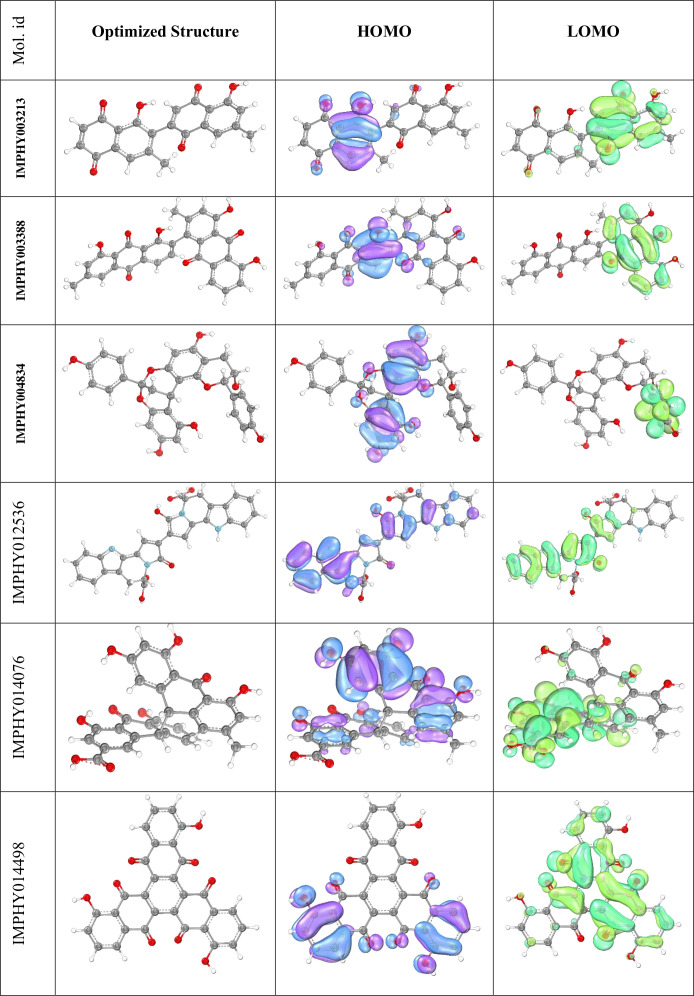
Density Functional Theory (DFT) analysis results for top-ranked compounds. For each molecule: (A) optimized molecular structure obtained using ORCA software with B3LYP/6-31G(d,p) basis set, (B) Highest Occupied Molecular Orbital (HOMO) distribution shown in blue/purple indicating electron-rich regions available for bonding, and (C) Lowest Unoccupied Molecular Orbital (LUMO) shown in green representing electron-deficient areas capable of accepting electrons. Electronic properties calculated include HOMO-LUMO gap, ionization potential, electron affinity, and dipole moment for drug-target interaction prediction.

DFT is used to predict the electronic structure properties of molecules, gain insights into their chemical reactivity and physical properties, and to predict the molecular interactions and energetics of potential drug compounds with biological targets. The optimized structure, HOMO, and LOMO of all the molecules have been shown in Fig 3. The HOMO diagram illustrates the distribution of the highest-energy electrons in the molecule, which can interact with the biological target. The LUMO diagram represents where the molecule can potentially accept electrons.

After completion of comparative DFT Calculation of all the molecules, the HOMO, LUMO, HOMO-LUMO Gap, Ionization Potential, Electron Affinity, Electronegativity, Chemical Potential, Global Hardness, Global Softness, Electrophilicity Index, and Dipole Moment for all the molecules have been calculated and shown in Table 3.

**Table 3:** Comparative DFT Analysis of Candidate Molecules.

Property	IMPHY003213	IMPHY003388	IMPHY004834	IMPHY012536	IMPHY014076	IMPHY014498
HOMO (eV)	−0.2385	−0.2227	−0.1974	−0.1839	−0.217	−0.2401
LUMO (eV)	−0.1261	−0.1005	−0.0076	−0.1083	−0.0843	−0.1092
HOMO-LUMO Gap (eV)	0.1124	0.1222	0.1898	0.0756	0.1327	0.1309
Ionization Potential (IP, eV)	0.2385	0.2227	0.1974	0.1839	0.217	0.2401
Electron Affinity (EA, eV)	0.1261	0.1005	0.0076	0.1083	0.0843	0.1092
Electronegativity (χ, eV)	−0.1823	−0.1616	−0.1025	−0.1461	−0.15065	−0.17465
Chemical Potential (μ, eV)	0.1823	0.1616	0.1025	0.1461	0.15065	0.17465
Global Hardness (η, eV)	0.0562	0.0611	0.0949	0.0378	0.06635	0.06545
Global Softness (σ, eV ⁻ ¹)	17.7931	16.3666	10.5374	26.455	15.07156	15.278838
Electrophilicity Index (ω, eV ⁻ ¹)	0.29566	0.2137	0.05535	0.28234	0.171028	0.2330223
Dipole Moment (Debye)	7.56482	1.82226	3.7444	6.02785	3.23369	0.50868

Upon evaluating the electronic properties, five molecules emerge as promising for further study due to their electronic attributes, which suggest favorable reactivity and stability profiles. Molecule IMPHY003213 exhibits a substantial dipole moment, which implies strong potential for interactions in polar environments, despite a lower HOMO-LUMO gap, which may suggest increased reactivity. IMPHY003388, with its moderate dipole moment and higher HOMO-LUMO gap, offers a balance of reactivity and stability. IMPHY004834 stands out with the highest HOMO-LUMO gap, indicative of stability, yet its exceptionally low electron affinity may limit its reactivity. On the other hand, IMPHY014498, while displaying significant stability suggested by a high HOMO-LUMO gap, has the lowest dipole moment, potentially reducing its interaction with polar biological environments. IMPHY012536’s high global softness points to substantial reactivity, coupled with a high dipole moment for interaction potential. Lastly, IMPHY014076 presents a balanced profile with a good HOMO-LUMO gap and a reasonable dipole moment. Considering these factors, IMPHY003213, IMPHY003388, IMPHY012536, and IMPHY014076 emerge as the molecules with the most favorable electronic properties for further investigation in the realm of drug design and discovery.

### 3.4. Molecular dynamics simulation

To get critical insights into the dynamic behavior of protein-ligand complexes over time and the impact of ligand binding on proteins’ structural stability and the dynamic interaction between protein-ligand, a comprehensive 300 ns MD simulation has been conducted. All the MD trajectories have been thoroughly analyzed. This extensive analysis encompasses a detailed evaluation of parameters such as RMSD, RMSF, Rg, SASA, PCA, FEL, and hydrogen bond formation.

#### 3.4.1. RMSD.

**Fig 4:**
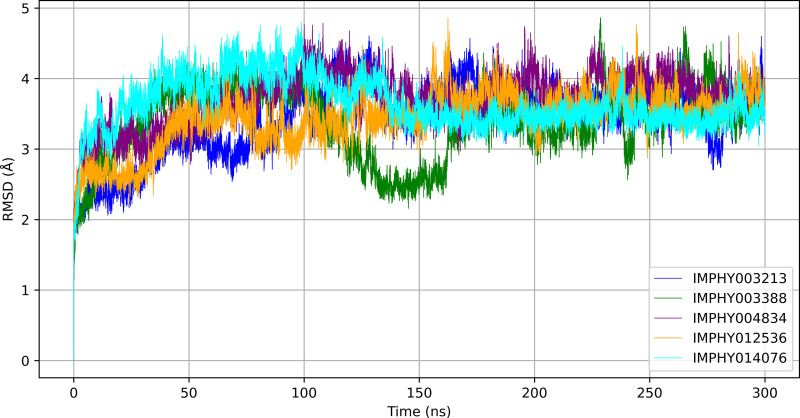
Comparative RMSD trajectories of five ligand-bound protein complexes across a 300 ns simulation period.

The RMSD analysis over a 300 ns MD simulation reveals the temporal dynamics of protein-ligand complexes, as displayed in Fig 4.

IMPHY003213’s RMSD fluctuated throughout the simulation, with an average of 3.428 ± 0.501 Å. It exhibited notable peaks in RMSD at around 50–100 ns and 250 ns, suggesting transient conformational changes. IMPHY003388 showed a heavily fluctuating RMSD throughout the simulation, averaging 3.394 ± 0.518 Å. The IMPHY004834 complex exhibited the highest level of conformational flexibility, with an average RMSD of 3.714 ± 0.368 Å, characterized by significant deviations, particularly within the first 50 ns. For IMPHY012536, the RMSD averaged 3.440 ± 0.400 Å, indicating moderate stability with occasional spikes in RMSD, suggesting episodic flexibility at approximately 100 ns and 225 ns. Lastly, IMPHY014076 exhibited an average RMSD of 3.641 ± 0.355 Å, with distinct fluctuations, particularly at 75 ns and 175 ns, indicating dynamic conformational shifts during the simulation period.

#### 3.4.2. RMSF.

**Fig 5:**
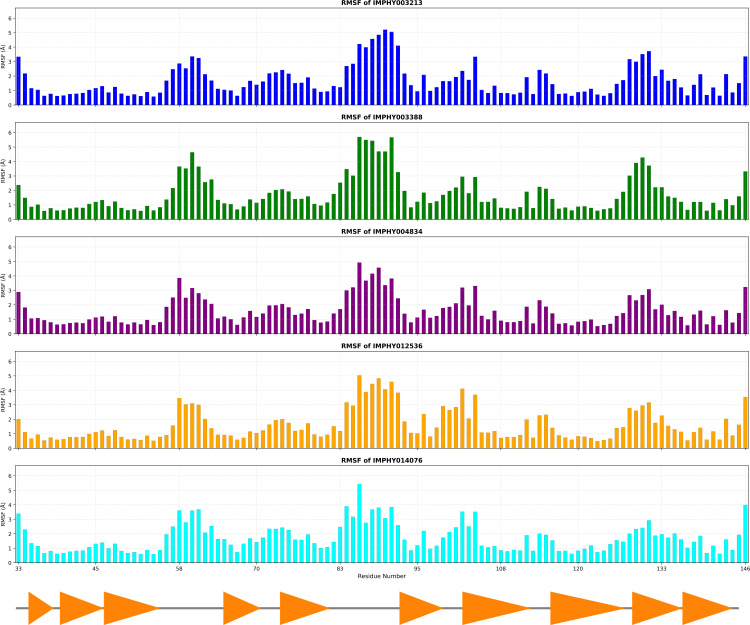
RMSF for Multiple Ligand-Bound Protein Complexes, highlighting local flexibility variations.

RMSF analysis over a 300 ns molecular dynamics simulation provides insights into the local flexibility of each residue in the protein when bound to various ligands. The RMSF bar graphs (Fig 5) for the five ligands indicate the degree of fluctuation each amino acid residue undergoes, suggesting areas of stability and flexibility within the protein structure.

IMPHY003213 shows an average RMSF of 1.707 ± 1.076 Å. The bar graph displays pronounced peaks across the protein, indicating regions of significant flexibility that may be crucial for functional conformational changes. IMPHY003388 has a slightly higher average RMSF of 1.758 ± 1.232 Å, which is the highest SD observed, suggesting this ligand induces the most fluctuation across the protein residues, potentially affecting the protein’s dynamic range. IMPHY004834 is associated with the lowest average RMSF of 1.601 Å and an SD of ±0.958 Å, indicating a more uniform and possibly more restrained conformational flexibility compared to the other ligands. IMPHY012536 exhibits an average RMSF of 1.613 ± 1.096 Å, indicating significant fluctuations in specific regions, which may correspond to functionally relevant sites for ligand binding or protein activity. IMPHY014076 has an average RMSF of 1.726 ± 0.971 Å, reflecting a diverse profile of residue flexibility, with specific areas being particularly mobile.

**Fig 6:**
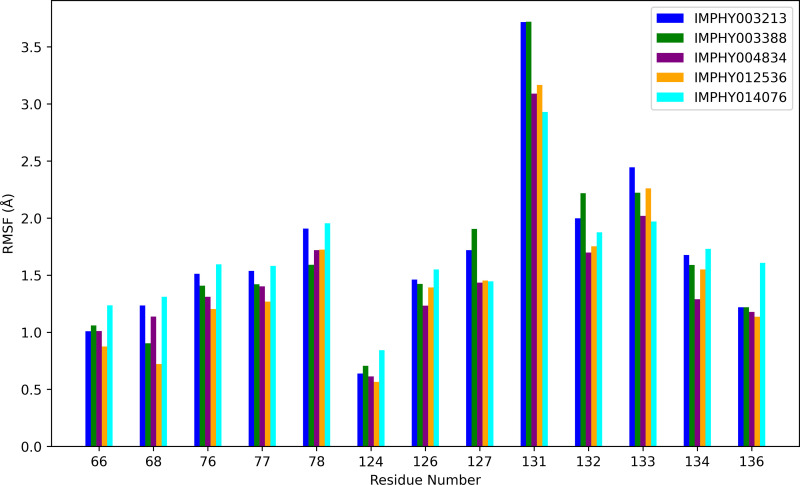
Comparative analysis of RMSF by residue number for distinct ligand-protein complexes, indicating differential local flexibility.

The RMSF bar chart (Fig 6) offers a focused comparison of residue flexibility for amino acid residues present at the active site of the protein structure when bound to different ligands. Specific residues, such as 126 and 131, exhibit notably high RMSF values across all ligands, indicating these regions are inherently flexible within the protein structure. IMPHY003213 and IMPHY003388 generally exhibit comparable levels of flexibility for most residues. They particularly influence residue 131, which shows some of the highest fluctuations, suggesting a potential impact on the conformational dynamics of the binding site. IMPHY004834 and IMPHY012536 exhibit a slightly lower RMSF for several residues but still contribute to significant fluctuations in residues 126 and 131, thereby maintaining the trend of heightened mobility at these sites. Such lower RMSF values in the presence of ligands could indicate a stabilizing effect of the ligand on the active site. IMPHY014076 tends to induce less fluctuation in specific residues, like 66 and 77, compared to other ligands, but it is consistent with the others in the high mobility seen in residues 126 and 131.

#### 3.4.3. Radius of gyration.

**Fig 7:**
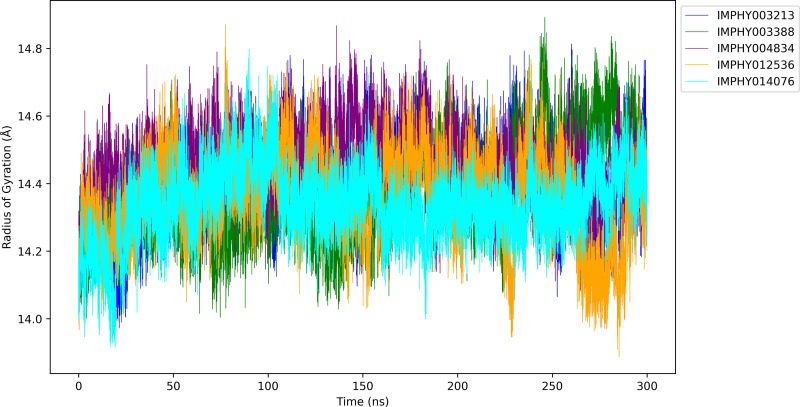
Comparative Rg trajectories of five ligand-bound protein complexes across a 300 ns simulation period.

The Radius of Gyration (Rg) plots (Fig 7) for each ligand-bound protein complex offer insight into the dynamic structural changes that occur over the 300 ns molecular dynamics simulation period:

The average of IMPHY003213 bound protein Rg is 14.395 ± 0.119 Å, indicating a relatively consistent structure with subtle fluctuations. However, there are moments, especially around the 150 ns and 250 ns marks, where noticeable deviations from the average occur, suggesting transient structural expansions. With an average Rg of 14.380 ± 0.138 Å, the IMPHY003388 complex exhibits slightly lower compactness. Significant expansions are observed, particularly around 100 ns and again between 200 ns and 250 ns, which may reflect more dramatic conformational rearrangements. Exhibiting the highest average Rg at 14.458 Å ± 0.098 Å, the IMPHY004834 complex maintains a less compact structure throughout the simulation. Notable is the period after 50 ns where the Rg briefly spikes, indicating a transient expansion before stabilizing into a consistent, more expanded state. IMPHY012536 complex has the lowest average Rg of 14.361 ± 0.131 Å, indicating a highly compact structure. The Rg plot shows a few notable peaks, particularly around 75 ns and 225 ns, but these are relatively small in magnitude, pointing to minor transient conformational shifts. The average Rg of IMPHY014076-bound PD1 is 14.340 ± 0.106 Å, implying a tightly compact protein conformation. While the structure remains stable in general, there are discrete instances of fluctuation, such as around 125 ns and again near 275 ns, which may indicate brief periods of loosening within the protein structure.

#### 3.4.4. Solvent accessible surface area.

**Fig 8:**
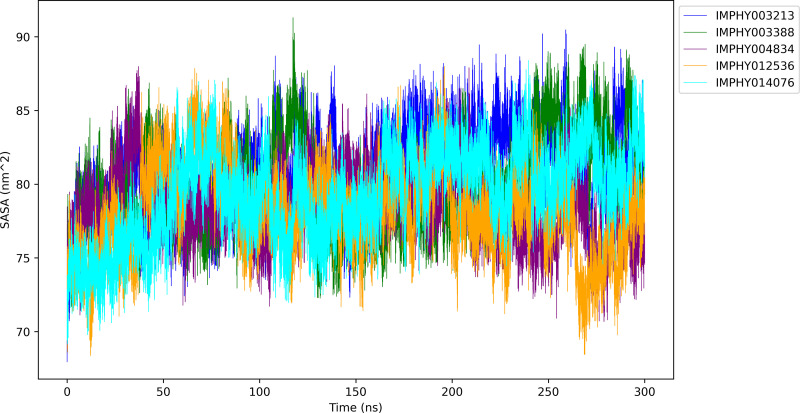
SASA Profiles of protein-ligand complexes over 300 ns Molecular Dynamics Simulations.

To assess the exposure of protein residues to the solvent over the course of the 300 ns simulation and get insights into the conformational behavior of the protein in the presence of various ligands, the SASA has been computed and displayed in Fig 8.

The IMPHY003213 complex exhibits a notable average SASA of 80.97 ± 2.76 nm², indicating a dynamic interaction with the solvent characterized by significant conformational shifts. Peaks in SASA are observed around 100 ns and 200 ns, suggestive of transient unfolding or exposure of the protein’s hydrophobic core. The IMPHY003388 complex displays an average SASA of 79.91 ± 3.06 nm², reflecting a more pronounced variability in solvent exposure. This may denote a flexible protein surface capable of adapting to different solvent environments or ligand-induced conformational states. The IMPHY004834 complex presents the lowest average SASA of 78.48 ± 2.32 nm², suggesting a more consistent and compact protein conformation with reduced solvent interaction. Despite the lower average, occasional increases in SASA are observed, indicating moments of greater solvent accessibility. The IMPHY012536 complex is characterized by a similar average SASA of 78.19 ± 2.97 nm², hinting at a tightly packed protein structure. This is intermittently disrupted by peaks indicating increased solvent accessibility, aligning with potential structural reconfigurations at specific intervals. The IMPHY014076 complex yields an average SASA of 79.34 ± 3.06 nm², which is among the highest variabilities observed, pointing to a dynamic surface that periodically transitions to more open conformations. Collectively, the SASA data indicate that the ligand-bound protein complexes differ in their degree of solvent exposure, with IMPHY004834 and IMPHY012536 associated with the most consistent and possibly more stable conformations. Conversely, the IMPHY003213 and IMPHY014076 complexes demonstrate a higher degree of conformational flexibility and dynamic solvent interactions. These variations in solvent accessibility and structural compactness across the protein-ligand complexes are crucial for understanding the molecular basis of ligand binding and the subsequent biological implications.

#### 3.4.5. Principal component analysis.

**Fig 9:**
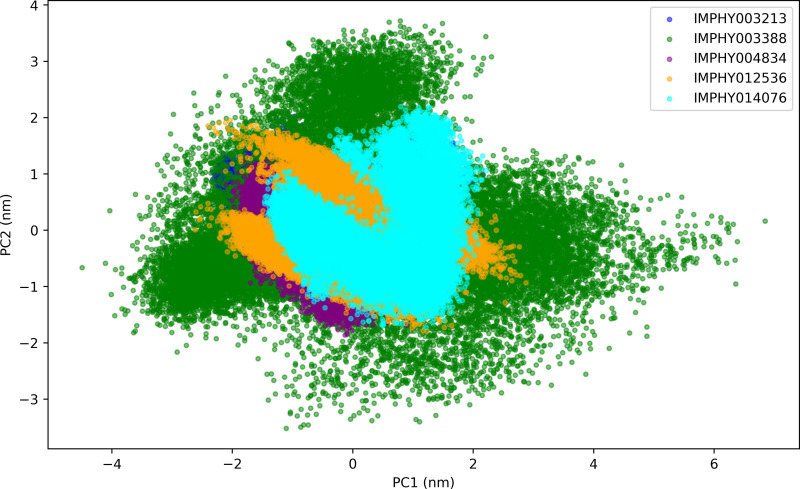
Distribution of protein conformational states mapped onto the first two principal components (PC1 and PC2) for a selection of ligand-bound complexes over a 300 ns simulation.

Principal Component Analysis (PCA) elucidates the conformational space sampled by protein-ligand complexes during a 300 ns molecular dynamics simulation. The PCA scatter plots reveal the range of motions along the two principal axes, PC1 and PC2, providing a comparative view of the flexibility and structural variety induced by different ligands shown in Fig 9.

The PCA of IMPHY003213-bound protein delineates a confined conformational space, with PC1 ranging from −2.327 to 2.166 nm and PC2 from −1.348 to 2.026 nm. This limited spread indicates specific, constrained protein movements when the protein is bound to this ligand. IMPHY003388 complex samples an expansive conformational space, as shown by a broad PC1 range from −4.494 to 6.849 nm and PC2 range from −3.521 to 3.716 nm. The wide distribution suggests a highly flexible protein conformation, capable of adopting diverse structural configurations. IMPHY004834 complex exhibits a narrower range for PC1 from −2.100 to 1.747 nm and for PC2 from −1.862 to 1.629 nm, indicating a more selective and potentially stable set of protein conformations under the influence of the ligand. The conformational spread for IMPHY012536-bound protein shows PC1 ranging from −2.615 to 2.878 nm and PC2 ranging from −1.793 to 1.950 nm. The asymmetry in the spread between the principal components suggests specific directional flexibility in the protein structure. The PCA of IMPHY014076 bound protein demonstrates a range for PC1 from −1.466 to 2.222 nm and for PC2 from −1.727 to 2.196 nm. The similarity in ranges for both components implies a balanced conformational variability influenced by the ligand. The PCA analysis presents a vivid picture of how ligand binding affects the conformational dynamics of the protein. Notably, IMPHY003388 enables the protein to explore the most extensive conformational range, indicating the potential for multiple binding or allosteric modulation pathways. Conversely, complexes with IMPHY004834 and IMPHY003213 maintain a more constrained conformational range, suggesting these ligands may lock the protein into more specific shapes.

#### 3.4.6. Free energy landscape.

**Fig 10:**
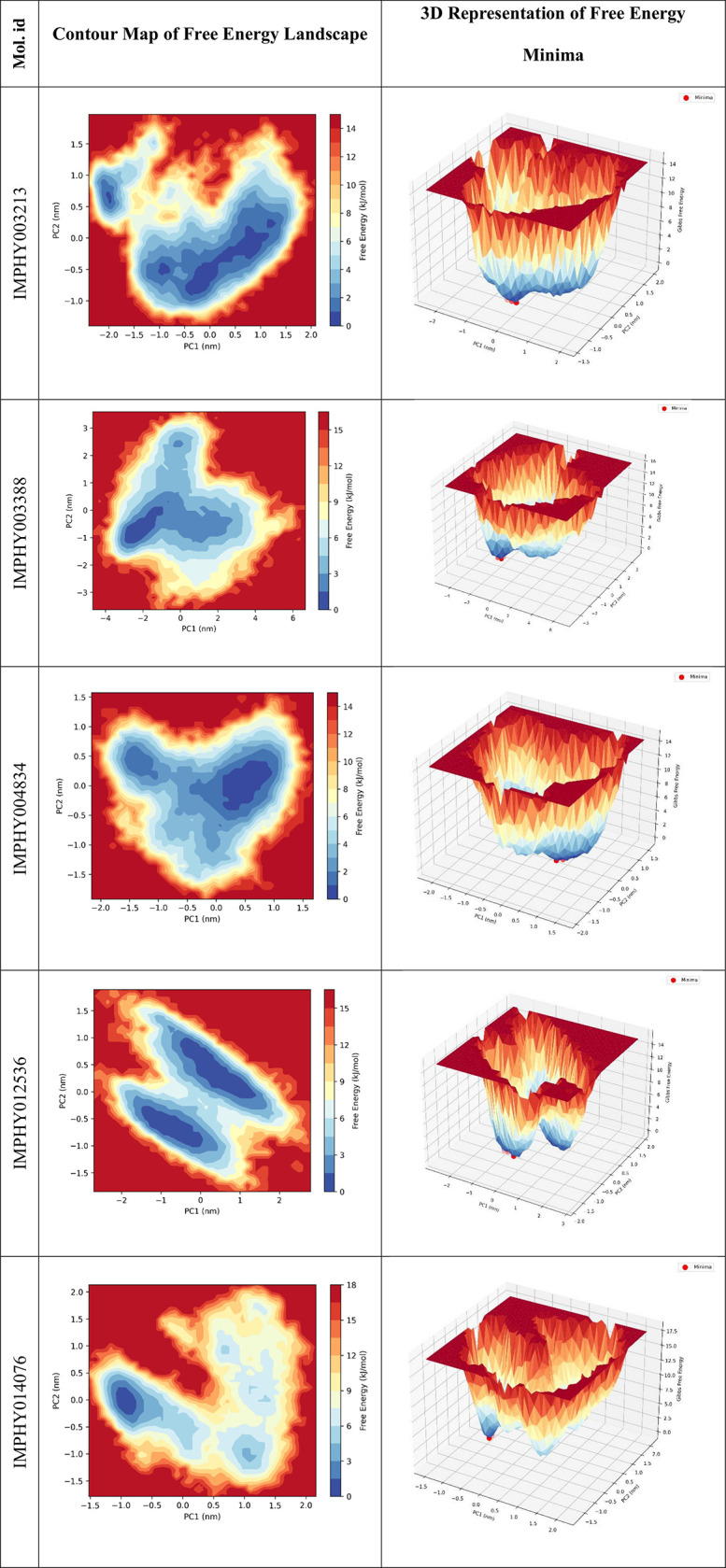
Contour and 3D Visualization of the Free Energy Landscape for all five ligand-bound proteins, Demonstrating Stable Conformational Minima and Transition States.

The free energy landscapes (FEL) of protein-ligand complexes provide a thermodynamic snapshot of the conformational states accessible during molecular dynamics simulations. The landscapes, represented by both contour maps and 3D visualizations, reveal the minima and the transitional barriers, reflecting the stability and diversity of protein conformations (Fig 10).

The FEL of IMPHY003213 bound protein exhibits a broad basin with multiple accessible minima, as depicted in the contour map. The corresponding 3D representation highlights the presence of several low-energy states, indicating that the protein exhibits multiple stable conformations when complexed with this ligand. The contour map for IMPHY003388 bound protein reveals a large, deep basin, indicating a singular, highly favored energy minimum. The 3D view shows a pronounced central well, highlighting a strong preference for one predominant conformation over others. IMPHY004834-bound protein presents a free energy landscape with a broad central trough, as seen in the contour map. The 3D landscape underscores this central low-energy region, flanked by higher energy barriers, implying a preference for a particular conformational range with potential for diverse, yet energetically similar, states. The contour map of IMPHY012536 bound protein showcases two distinct energy minima, separated by moderate energy barriers. The 3D representation confirms these findings, illustrating the protein’s potential to alternate between these two favored states. The complex of IMPHY014076 FEL features an asymmetric landscape with multiple minima, as shown in the contour map. The 3D visualization demonstrates these varied low-energy states, separated by distinct energy barriers, suggesting a dynamic conformational equilibrium within the protein. The comparison of free energy landscapes across these protein-ligand complexes elucidates the varied thermodynamic influences of different ligands. Specific ligands, such as IMPHY003388, seem to stabilize the protein in a particular conformation, whereas others, like IMPHY003213 and IMPHY014076, promote a spectrum of energetically favorable conformations. These insights are fundamental to our understanding of protein dynamics and can guide the optimization of ligand design for enhanced binding specificity and efficacy.

#### 3.4.7. Dynamics of hydrogen bond formation in protein-ligand complexes.

**Fig 11:**
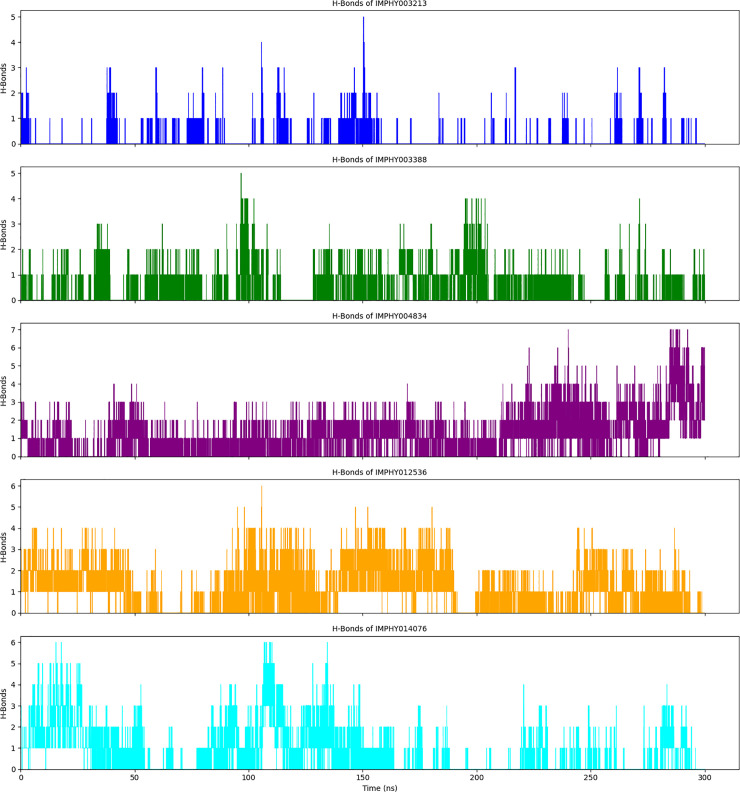
Temporal analysis of hydrogen bond counts in multiple ligand-protein complexes over 300 ns molecular dynamics simulations.

The quantitative analysis of hydrogen bond formation throughout a 300 ns molecular dynamics simulation reveals the temporal stability and interaction specificity of protein-ligand complexes (Fig 11). The number of hydrogen bonds, a key determinant of the strength and stability of the protein-ligand interactions, is monitored and compared across five different ligands.

The complex IMPHY003213 forms hydrogen bonds with a fluctuating pattern, indicating dynamic interactions with the active site. Throughout the simulation, there are intervals with increased numbers of hydrogen bonds, suggesting periods of tighter binding. Complex IMPHY003388 exhibits higher variability in hydrogen bond numbers, indicating a potentially more flexible binding mode. Spikes in the number of bonds are observed, which may correspond to transiently stronger interactions. Sustained hydrogen bonding in IMPHY004834 complex (2–3 bonds throughout simulation) indicates stable interaction maintenance, contrasting with fluctuating patterns in other complexes that suggest binding instability. This consistency suggests a stable interaction with the active site. IMPHY012536 complex exhibits a variable pattern in hydrogen bond formation, with specific periods showing an increased number of bonds. These periods may be indicative of structural conformations that are more conducive to binding. Complex IMPHY014076 shows distinct fluctuations in hydrogen bond numbers over time. The wide range of values observed suggests that the ligand may induce various conformational states within the protein, collectively, with some complexes, such as IMPHY004834, to more stable interactions and others to more dynamic binding modes.

#### 3.4.8. Free binding energy with MM-PBSA.

The molecular mechanics Poisson-Boltzmann surface area (MM-PBSA) calculations provided a quantitative assessment of binding thermodynamics for the top-ranked phytochemical candidates (Table 4). IMPHY004834 demonstrated the most favorable binding affinity with a free energy of −85.302 ± 10.291 kJ/mol. IMPHY012536 exhibited the second-strongest binding affinity, followed by IMPHY003388.

**Table 4:** MM-PBSA binding free energy analysis.

Molecule id	van der Waal energy (kJ/mol)	Electrostatic energy (kJ/mol)	Polar solvation energy (kJ/mol)	SASA energy (kJ/mol)	Binding energy (kJ/mol)
IMPHY003213	−18.245 ± 0.025	−21.340 ± 0.348	−16.890 ± 13.767	−3.548 ± 0.312	−60.023 ± 13.775
IMPHY012536	−38.488 ± 2.091	2.078 ± 3.454	−24.725 ± 3.987	−4.597 ± 0.439	−65.732 ± 5.691
IMPHY004834	−66.150 ± 3.326	−113.386 ± 6.462	114.796 ± 7.282	−20.562 ± 0.264	−85.302 ± 10.291
IMPHY003388	−76.022 ± 3.549	−26.748 ± 2.482	48.358 ± 5.332	−8.602 ± 0.722	−63.014 ± 6.907
IMPHY014076	−16.120 ± 0.014	−18.840 ± 0.807	−14.680 ± 0.682	−3.333 ± 0.142	−52.973 ± 1.066

#### 3.4.9. Integrated analysis and ranking for in vitro validation.

Based on a comprehensive 300 ns MD simulation analysis, we established the following stability and equilibrium criteria for PD-1 inhibitor selection: **Stability Assessment and Equilibrium State Analysis.**

**Stability Assessment: (i) RMSD stability** (Complexes with average RMSD < 3.5 Å and minimal fluctuations indicate stable binding conformations). **(ii) RMSF consistency** (Lower RMSF values (< 1.65 Å) suggest reduced local flexibility and more stable interactions). **(iii) Structural compactness** (Consistent Rg values indicate maintained protein structural integrity). **(iv) Binding affinity** (MM-PBSA binding energies < −60 kJ/mol indicate strong protein-ligand interactions).

**Equilibrium State Analysis:** The equilibrium state was defined as the period after 100 ns, during which structural parameters remained within ±10% of their mean values, indicating Stable protein-ligand complex formation, no further major conformational rearrangements, and Sustained interaction networks. Table 5 shows the ranking for *In Vitro* Validation.

**Table 5:** Ranking for *In Vitro* Validation.

Rank	Molecule ID	Stability Score*	Key Advantages	In Vitro Priority
1	**IMPHY004834**	9.2/10	Lowest RMSF (1.601 Å), highest binding affinity (−85.302 kJ/mol), consistent H-bonds	**HIGH**
2	**IMPHY012536**	8.7/10	Most compact structure (Rg: 14.361 Å), strong binding (−65.732 kJ/mol)	**HIGH**
3	**IMPHY003388**	7.5/10	Good binding (−63.014 kJ/mol), moderate stability	**MEDIUM**
4	**IMPHY003213**	7.2/10	High uncertainty in binding, variable H-bonding	**MEDIUM**
5	**IMPHY014076**	6.8/10	Lowest binding affinity (−52.973 kJ/mol)	**LOW**

**Fig 12:**
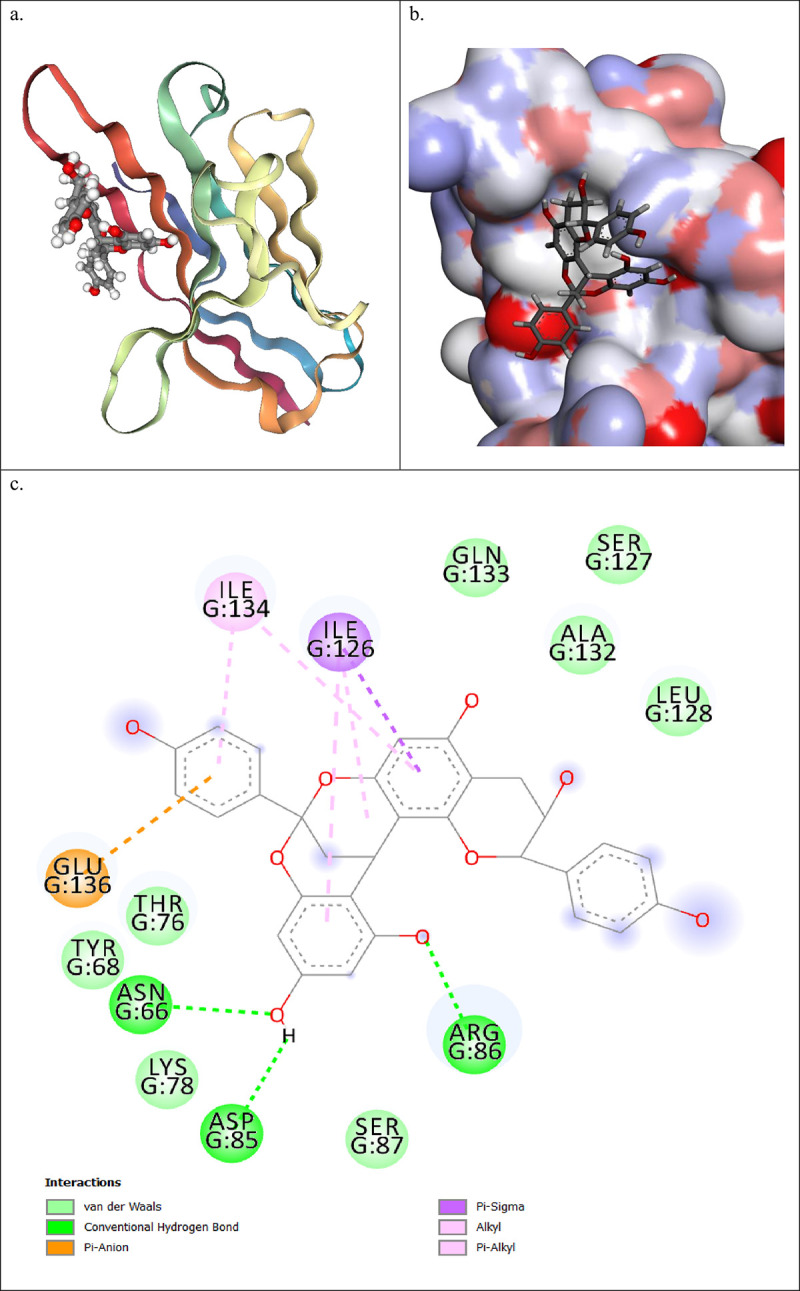
Comprehensive Visualization of IMPHY004834-PD-1 Interaction: (a) 3D Ribbon Model (b) Surface Electrostatic Potential Map, and (c) Detailed Interaction Schematic Highlighting Specific Residue Contacts and Binding Modes.

**Fig 13:**
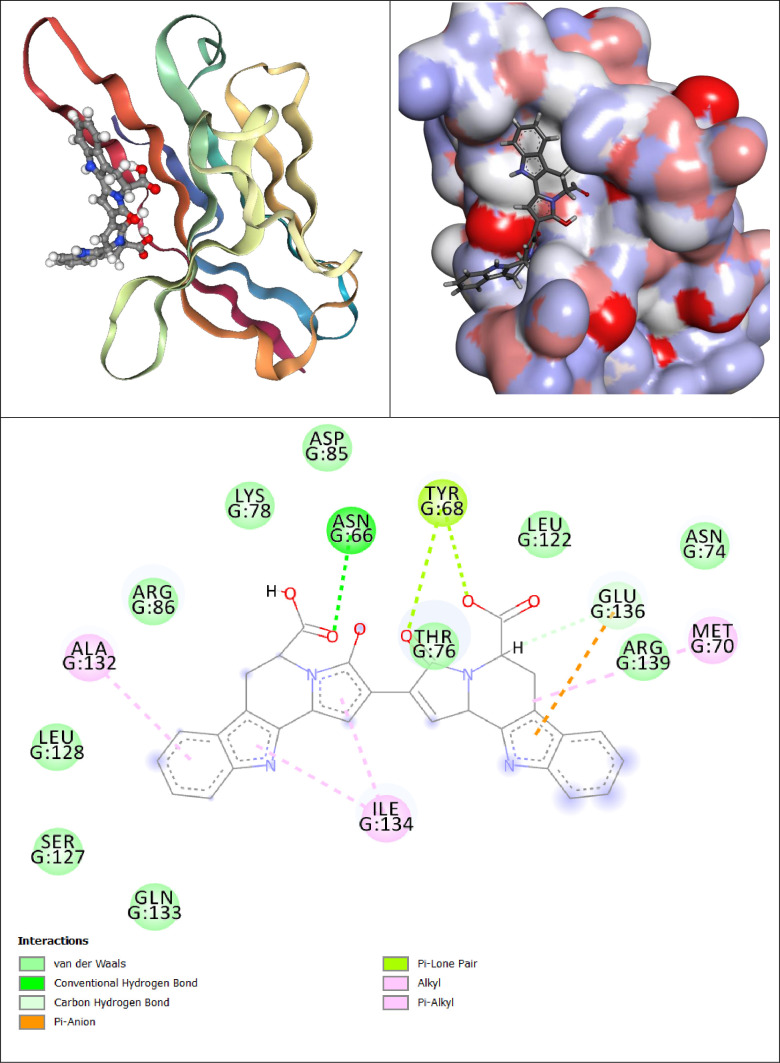
Comprehensive Visualization of IMPHY012536-PD-1 Interaction: (a) 3D Ribbon Model, (b) Surface Electrostatic Potential Map, and (c) Detailed Interaction Schematic Highlighting Specific Residue Contacts and Binding Modes.

Stability score based on a weighted combination of RMSD consistency (25%), RMSF (25%), binding energy (30%), and H-bond stability (20%). Based on the detailed MD simulation trajectory analysis, IMPHY004834 stands out due to its consistent and stable interaction with the protein, as indicated by its lowest average RMSF and relatively stable RMSD profile. The consistent number of hydrogen bonds throughout the simulation underscores a stable interaction with the active site. Additionally, its Free Energy Landscape (FEL) reveals a preference for a particular conformational range, suggesting a specific and stable binding mode. This ligand exhibits a balanced profile that maintains both structural integrity and flexibility, which are crucial for effective binding and potential inhibitory activity. IMPHY012536 presents as another notable candidate, characterized by its compact structure (lowest Rg) and moderate stability in RMSD analysis. Its FEL showcases two distinct energy minima, suggesting the protein’s ability to alternate between favored states, potentially enhancing the ligand’s adaptability and efficacy. Despite its variable pattern in hydrogen bond formation, periods of increased bonding may indicate strong interactions conducive to binding. IMPHY004834 exhibits remarkable stability and a potentially more specific interaction with the active site, making it a prime candidate for further investigation. Its consistent hydrogen bond formation and lower RMSF values indicate a stable and specific binding, which could translate into effective inhibitory activity. IMPHY012536, while showing episodic flexibility and a compact structure, suggests adaptability through its dual energy minima in FEL analysis. This could be advantageous for engaging with dynamic protein conformations; however, further investigation may be necessary to understand its binding dynamics fully. Thus, IMPHY004834 emerges as the most promising molecule due to its stability, specificity, and favorable energetic profile. IMPHY012536 also shows potential, particularly in terms of structural adaptability and compactness, warranting its inclusion for further detailed study and validation. The 2D and 3D binding modes for both ligands have been shown in Figs 12 and 13.

Fig 12 shows that the aromatic rings in IMPHY004834, which are electron-rich as evidenced by the localization of the HOMO, enable pi-sigma interactions with the isoleucine residues ILE126 and ILE134. These interactions are facilitated by the delocalized electrons in the aromatic system, which can overlap with the sigma orbitals of the hydrophobic side chains of these amino acids, thereby anchoring the ligand within the protein’s binding site. The alkyl groups engage in hydrophobic interactions with the side chains of LEU128 and ALA132. The nonpolar nature of alkyl chains complements the hydrophobic environment provided by these residues, facilitating van der Waals forces that are fundamental for my binding stability. Furthermore, the architecture of IMPHY004834 allows for pi-alkyl interactions with GLN133, where the electron cloud of aromatic rings interfaces with the alkyl side chains of glutamine, enriching the tapestry of hydrophobic interactions that enhance my binding affinity. The presence of hydrogen bond donors and acceptors in the molecular framework, such as polar functional groups: hydroxyl and amide groups, permits IMPHY004834 to form conventional hydrogen bonds with TYR68, THR76, ASN66, and LYS78. These hydrogen bonds are critical for IMPHY004834’s precise orientation and for maintaining a strong and specific interaction with the protein. Lastly, HOMO and LUMO visualizations suggest that the charged moieties, particularly carboxylate groups, engage in pi-anion interactions with GLU136. This interaction arises due to the electrostatic attraction between the negatively charged oxygen atoms of the ligand and the positively charged regions near the glutamate residue.

Fig 13 shows that the IMPHY012536 facilitates van der Waals interactions with LEU G:128 and ILE G:134 through the alkyl chains in my framework, engaging these hydrophobic side chains in stabilizing contacts. This interaction is likely influenced by the areas of the structure that lack prominent electronic features, making them ideal for close-contact hydrophobic interactions. The electron-rich area of HOMO on the ligand is positioned to act as hydrogen bond donors or acceptors and form strong directional interactions with THR G:76 and TYR G:68. The carbon-hydrogen bond with ASN G:66 involves slightly acidic hydrogens, which could be associated with the areas of slight electron density in the HOMO, indicating the ligand’s ability to interact through weaker hydrogen bonds with electron-rich donors like the side chain of asparagine. The LUMO of IMPHY012536 reveals electron-deficient areas that are poised for pi-anion interactions with negatively charged amino acids, such as GLU G:136. These interactions are indicative of regions on the ligand that can accept electron density from electron-rich donors, such as the carboxylate side chain of glutamate. Additionally, pi-alkyl interactions with LEU G:122 are supported by the geometry of the ligand’s aromatic rings, which, while not directly involved in bonding interactions as shown by the LUMO, can induce stabilizing van der Waals forces with hydrophobic side chains due to the shape and presence of the pi cloud. The pi-lone pair interaction with ARG G:139 suggests an interaction between the electron-rich lone pair on arginine and the electron-deficient areas of the ligand, as indicated by the LUMO.

### 3.5. ADME

The ADME properties of drugs significantly impact drug development, and global regulatory agencies play a key role in guiding pharmacogenomics during the early stages. Thorough characterization and in-depth ADME study are crucial for safer and more effective biotherapeutics [85–87]. The ADME properties of both molecules have been calculated and given in the supplementary material (Suppl2-swissadme.csv). The ADME properties of IMPHY004834 exhibit low GI absorption and water solubility, and it is an inhibitor of CYP2C9 and CYP3A4. Therefore, this molecule should not be administered orally but rather via a parenteral route. Due to no PAINS alert, the molecule does not seem to be showing any promiscuous or off-target binding. The IMPHY012536 exhibits low gastrointestinal absorption and poor water solubility, with P-glycoprotein substrate characteristics, inhibits CYP2C9, and has no PAINS alert. These features indicate potential challenges in oral bioavailability, and the PAINS alert suggests the possibility of non-specific biological reactivity, warranting careful consideration in lead optimization. Based on ADME studies, IMPHY004834 seems to have better ADME properties than IMPHY012536. Thus, IMPHY004834 should be taken for further pre-clinical studies.

## 4. Discussion

The annual occurrence of melanoma and non-melanoma skin cancer cases is steadily increasing [88]. In contrast to currently available treatment modalities, such as chemotherapy, radiation, and surgery, immunotherapy has substantially improved SCC patients’ survival rates and overall quality of life [89]. T-cells become activated and interact with tumor cells that have an excessive amount of PD-L1 [90]. This excessive PD-L1 binds with PD-1, which limits the T-cells’ capacity to initiate cell death. Monoclonal antibodies inhibit this interaction, and the use of either monotherapy or combination therapy has demonstrated promising results [90]. Currently, there are several phytochemicals available that block the PD-1/PD-L1 interaction by Inhibiting PD-L1 [91–95]. But, PD-L1 inhibitors only block the interaction between PD-1 and PD-L1; They do **not** block the interaction between PD-1 and its other ligand, PD-L2. As a result, tumor cells can still suppress immune responses via the PD-1/PD-L2 axis, potentially allowing immune escape and reducing therapeutic efficacy [96]. Whereas PD-1 inhibitors block the PD-1 receptor on T cells, preventing its interaction with both PD-L1 and PD-L2; this results in a more comprehensive release of immune inhibition, potentially leading to better antitumor responses [96]. Since 2014, numerous PD-1/PD-L1-based monoclonal antibodies have been developed, and various drug repurposing methodologies have been introduced to enhance drug discovery [97]. Various factors, including immunological considerations and the inadequate permeability of tumor tissues, often limit the efficacy of PD-1/PD-L1 antibody therapies. In contrast, small-molecule inhibitors have demonstrated greater efficacy compared to monoclonal antibodies in addressing these concerns [98]. Hence, identifying small molecule inhibitors that selectively target the PD-1/PD-L1 signaling pathway is of utmost importance to augment tumor immunotherapy. Peptides and small-molecule inhibitors have often been explored to target the PD-1/PD-L1 interaction [99–101]. Recently, several preclinical-level studies have been conducted [98,102–108] to identify small molecule-based alternatives to inhibit PD-1 and PD-L1 interactions. Our work could be another effort in the search for blocking the PD-1/PD-L1 interaction using natural product-based small molecules.

The integrated MD analysis conclusively demonstrates that IMPHY004834 (Mahuannin D) represents the most promising PD-1 inhibitor candidate based on multiple convergent stability metrics such as Achieved equilibrium state within 50 ns with sustained stability, Demonstrated the strongest binding affinity (−85.302 kJ/mol) via MM-PBSA, Maintained the most stable local conformations (RMSF: 1.601 Å), Formed consistent hydrogen bond networks throughout simulation, Exhibited optimal structural compactness without significant drift. These findings indicate that Mahuannin D adopts a stable, high-affinity binding conformation with PD-1, suggesting minimal conformational entropy loss upon binding and reduced likelihood of dissociation under physiological conditions. IMPHY012536 emerges as the secondary candidate, with exceptional structural compactness and a strong binding affinity, warranting further investigation. The stability profiles strongly support prioritizing these compounds for Synthesis and purification, and *in-vitro* studies*.* Mahuannin D is a flavonoid type (Subclass: Biflavonoids and polyflavonoid) phytochemical found in *Ephedra sinica* [109]. The cytotoxic effect of Mahuannin D has already been reported; however, the mechanism of action remains unknown. This is the first time we have attempted to establish that Mahuannin D can form a proper binding to PD-1 through this study. Due to the strong interaction between Mahuannin D and PD-1, PD-L1 is unable to bind to PD-1, allowing T cells to attack cancer cells and exhibit cytotoxicity. Thus, Mahuannin D should be taken for further lead optimization so that natural product-inspired PD-1 inhibitors could be developed that may overcome the limitations associated with Mahuannin D.

## 5. Conclusion

This comprehensive computational investigation successfully identified IMPHY004834 (Mahuannin D) from Ephedra sinica as a highly promising natural product-derived PD-1 inhibitor through systematic virtual screening of 17,967 phytochemicals. The integrated analytical approach, encompassing consensus molecular docking, 300-ns molecular dynamics simulations, DFT calculations, and ADME profiling, established multiple convergent lines of evidence supporting the therapeutic potential of Mahuannin D. Quantitatively, IMPHY004834 demonstrated exceptional binding affinity (−85.302 ± 10.291 kJ/mol via MM-PBSA), superior structural stability (lowest RMSF: 1.601 Å), and sustained interaction networks throughout extended simulations. The biflavonoid structure exhibits optimal electronic properties (HOMO-LUMO gap: 0.1898 eV) and forms critical interactions with key PD-1 residues (Tyr68, Thr76, Asn66, Lys78, Ile126, Ile134), establishing a stable binding conformation that effectively blocks PD-L1 engagement. This work addresses critical therapeutic gaps by targeting PD-1 directly rather than PD-L1, providing broader immune checkpoint blockade that prevents escape via the PD-1/PD-L2 axis. Unlike current monoclonal antibody therapies, small-molecule inhibitors offer several advantages, including potential for oral bioavailability, enhanced tissue penetration, reduced manufacturing costs, and simplified logistics. The identification of Mahuannin D represents the first evidence for its PD-1 inhibitory mechanism, expanding its known cytotoxic effects into immune checkpoint modulation. This discovery establishes a validated computational framework for natural product-based immune checkpoint inhibitor discovery and provides strong rationale for experimental validation including synthesis, in vitro PD-1/PD-L1 blockade assays, cell-based immune activation studies, and pharmacokinetic optimization. Future research directions include structural optimization to enhance oral bioavailability, experimental validation of predicted Ki values, investigation of synergistic combinations with existing therapies, and exploration of broader applications across PD-1-relevant malignancies. This work contributes to the growing paradigm of precision natural product drug discovery for next-generation cancer immunotherapy.

### Declaration

The English language quality of this article has been improved through the utilization of LLMs, specifically Llama-2-70B-GGML and Chat Generative Pre-trained Transformer 4.0 from OpenAI. The complete liability for the content of this publication rests with us. A thorough examination, refinement, and development have been implemented on all languages produced by the LLM to guarantee their exact correspondence with the results and interpretations of our initial research.

## Supporting information

S1 Material Results of Structure-based virtual screening PD1 against IMPAAT database. (CSV)

S2 Material ADME results (obtained form SwissADME). (CSV)

## Abbreviations

AADS: Automated Active Site identification
ADME: Absorption, Distribution, Metabolism, Excretion
CASTp: Computed Atlas of Surface Topography of proteins
CGenFF: CHARMM General Force Field
cSCC: cutaneous squamous cell carcinoma
DFT: Density Functional Theory
EGFR: Epidermal Growth Factor Receptor
FEL: Free Energy Landscape
HNSCC: head and neck squamous cell carcinoma
HOMO: Highest Occupied Molecular Orbital
irAEs: immune-related adverse events
LINCS: Linear Constraint Solver
LUMO: Lowest Unoccupied Molecular Orbital
MDAnalysis: molecular dynamics analysis toolkit
MM-GBSA: Molecular Mechanics Generalized Born Surface Area
MM-PBSA: Molecular Mechanics Poisson-Boltzmann Surface Area
NPT: Number of particles, Pressure, and Temperature ensemble
NSCLC: Non-Small Cell Lung Cancer
NVT: Number of particles, Volume, and Temperature ensemble
PAINS: Pan Assay Interference Compounds
PCA: Principal Component Analysis
PD-1: Programmed Death-1
PD-L1: Programmed Death-Ligand 1
PD-L2: Programmed Death-Ligand 2
PDBQT: Protein Data Bank, Partial Charge (Q), & Atom Type (T)
PME: Particle Mesh Ewald
QSAR: Quantitative Structure-Activity Relationship
RbR: Rank-by-Rank
RBD: Receptor Binding Domain
Rg: Radius of Gyration
RMSD: Root Mean Square Deviation
RMSF: Root Mean Square Fluctuation
SASA: Solvent Accessible Surface Area
SBVS: Structure-Based Virtual Screening
SCC: Squamous cell carcinoma.
